# A Study on Gel/Space Ratio Development in Binary Mixture Containing Portland Cement and Meta-Illite Calcined Clay/Rice Husk Ash

**DOI:** 10.3390/gels8020085

**Published:** 2022-01-28

**Authors:** David O. Nduka, Babatunde J. Olawuyi, Opeyemi O. Joshua, Ignatius O. Omuh

**Affiliations:** 1 Department of Building Technology, College of Science and Technology, Covenant University, Ota 112233, Nigeria; opeyemi.joshua@covenantunivesity.edu.ng (O.O.J.); ignatius.omuh@covenantuniversity.edu.ng (I.O.O.); 2 Department of Building, School of Environmental Technology, Federal University of Technology, Minna 920211, Nigeria; babatunde@futminna.edu.ng

**Keywords:** gel/space ratio, meta-illite calcined clay, rice husk ash, Powers’ model, high-performance concrete, superabsorbent polymers

## Abstract

Supplementary cementitious materials (SCMs) have been widely used to enhance both the microscopic and macroscopic properties of the Portland cement (PC)–SCM composite matrix. Few studies have been undertaken to establish the gel/space ratio of meta-illite calcined clay (MCC) and rice husk ash (RHA)-based high-performance concrete (HPC) mortar. This experimental paper describes a conventional degree of hydration (non-evaporable water) and porosity routes of establishing a link amid the gel/space ratio and compressive strength of a sieved mortar from Class 1 (50–75 MPa) HPC at an early age. Using the non-evaporable water method, this paper predicted the gel/space ratio of the hardened MCC/RHA-based HPC mortars and curved fitted into Powers’ exponent equation. The results from this study revealed that MCC or RHA additions (5–30% by weight of PC) to the PC-SCM matrix led to a moderate decline in the compressive strength of the low water-binder ratio (W/B) HPC mortar. The modification aimed at void volume (superabsorbent polymers, SAP, and air) applying Bolomey’s formula and Powers’ gel/space ratio developed a suitable fitting into the Powers’ model. This experimental procedure shows feasibility to predict the MCC and RHA outcome on the compressive strength of HPC.

## 1. Introduction

In the framework of the continuous attempt to mitigate anthropogenic carbon dioxide (CO_2_) emission from cement industries, scholars and practitioners are paying attention to the inclusion of supplementary cementitious materials (SCMs) as total or partial replacement of cement in concrete and mortar. SCMs are mostly siliceous/aluminous finely segregated dense crystals that react with cement composite chemically and deplete calcium hydroxide to form a more cementitious product [1]. Improved mechanical, durability, and microstructural properties have been reported for SCM-based concrete and mortar [2,3,4]. The production of concrete is influenced by many factors, such as strength, workability, water-binder ratio (W/B), and durability targets which will be uncertain until a specific application is established. These factors are pointers to the class or performance criteria of concrete to be developed. In Nigeria, the blend of Portland cement (PC) with SCMs is rare in concrete and mortar for several reasons. These may be associated with the high cost of production, unavailability of SCMs, early age cracking potentials, and ultimately, lack of awareness by prominent professionals in the built environment [5].

In promoting the addition of SCMs in mortar and concrete either fully or partially, the Nigerian government, through the Nigerian Building and Road Research Institute (NBRRI), an organ of the Federal Ministry of Science and Technology, in 2015 installed and commissioned a 2-tonne/day capacity pilot calcined clay based pozzolan cement plant in Ota, Ogun State, Nigeria [6]. Olawale et al. [7] partially replaced PC with calcined clay-based NBRRI pozzolan and glass fibre-reinforced samples and characterised the mechanical properties. NBRRI pozzolan was varied between 3–15% with a concrete design mix of 1:1.5:3 ratio at a constant W/B of 0.45. Their results showed that the concrete’s workability reduced with the increases in the percentage of NBRRI cement. They also obtained best strength values of 38.99 N/mm^2^, 3.5 N/mm^2^, and 6.12 N/mm^2^, respectively, for compressive, split tensile, and flexural strengths at 12% blend calcined clay. The results were adjudged consistent with the mechanical properties of normal strength concrete (NSC).

NSC is a major material problem in heavy civil engineering projects. It results in low strength and workability and durability issues in some specific applications, such as high-rise buildings, long-span bridges, pavements etc. [8]. NSC concrete contains PC, fine aggregate, coarse aggregate, and water. Oyebisi et al. [9] stressed that structural elements constructed with NSC in the 20th and 21st centuries deteriorate within the first 20 years of construction. To address the NSC shortcomings, high-performance concrete (HPC), usually blended with SCM with an optimised mix design, is usually adopted to improve the structural elements property. Specifically, HPC is linked with low porosity and high mechanical, durability, and microstructural properties. The mechanical properties of HPC are of great importance to designers and researchers in determining the engineering properties of hardened concrete. The importance of these properties could establish the HPC’s ability to resist failure under compressive forces, tensile forces, and deformation resistance capacity of concrete to meet the design requirements [10]. Compressive strength, tensile strength, modulus of elasticity, Poisson’s ratio, bond strength, and modulus of rapture are regarded as short-term mechanical properties of hardened concrete. However, compressive strength mostly governs the concrete design and material proportioning (whether normal, high-strength, or high-performance) among these macroscopic properties of the cement-SCM composite matrix. This property constantly transforms with the hydration of the cement and the emergence of the hydration outcomes [11]. Consequently, the cement reaction scale is an easy factor in explaining the microscopic progressions and it can calculate macroscopic variations, particularly in compressive strength.

Researchers view that equations and models used for NSC cannot be used or applied in HPC. The reasons given by Aitcin [12] are that HPC behaviour, especially in compression, is distinct from NSC, which has inspired new models from various studies for compressive strength, elastic modulus, and stress-strain behaviour. In addition, the original volume of water, cement, and air influence the compressive strength. In this regard, early porosity has been proposed to augment the connection between the cement reaction level and the compressive strength [11]. Wu et al. [11] also averred that cement reaction degree correlates to compressive strength once the early porosity for various paste equates. The studies of Powers and Brownyard [13] and Powers [14] first established the correlation concerning the compressive strength of concrete and gel/space ratio. Their exponent based model is as shown here in Equations (1) and (2).

*f_c_* = AX^n^ (1)

(2) $$X=\frac{\mathrm{volume}\;\mathrm{of}\;\mathrm{gel}}{\mathrm{volume}\;\mathrm{of}\;\mathrm{space}}$$

where A is the core strength of the material at nought porosity (MPa), X is the gel/space ratio, and n is an exponent. Powers found compressive strength (*f_c_*) values of A and n to be 234 MPa and 3, respectively. In their works, the degree of hydration at a particular cementitious material age determines concrete strength and gel/space ratio. Wu et al. [11] defined gel/space ratio in line with Powers and Brownyard [13] and Powers [14] “as the volume ratio of the gel to the available space in the composite system”. Later studies have investigated this link between gel/space ratio and compressive strength of cementitious materials in unary, binary, and ternary combinations of various SCMs [11,15,16,17,18]. Lam et al. [15] experimentally established a positive connection between gel/space ratio and compressive strength of binary high-volume fly ash (HVFA) cement paste and concrete. The separate cement and fly ash degree of hydration was first computed following the gel/space ratio estimation. Hasholt et al. [16] found a correlation among compressive strength and gel/space ratio on different concrete mixes containing different superabsorbent polymers (SAP) additions at different W/C ratios. The authors’ results complied with Powers and Brownyard [13] and Bolomeys [14] models.

Bolomeys [19] theorised that the effect of air voids generated by SAP on compressive strength could be credited in a similar approach as while contemplating the air content. Heikal et al. [17] included the association between gel/space ratio and compressive strength as parameters in determining the mechanical property of nano-alumina blended cement. Wu et al. [11] demonstrated the efficacy of ternary mixtures of cement, SF and FA, using the gel space ratio and compressive strength relationship. They found that the SCMs substitution accelerates gel/space ratio as the SCM degree of hydration reaction is higher at a certain calculated value. Moreover, Park and Choi [18] found a similar correlation between gel/space ratio and compressive strength in HVFA-based cement paste and mortar. Most studies have focused on the blend of fly ash, silica fume, and slag with PC in predicting the cement composite’s gel/space ratio. To the authors’ knowledge, the use of meta-illite calcined clay (MCC) and rice husk ash (RHA) SCMs is limited. Therefore, this study applied the PC and HVFA gel/space ratio and compressive strength relationship model by Lam et al. [15] to compute the link between the gel/space ratio and compressive strength of MCC/RHA-based HPC mortar and their fitting curves.

## 2. Materials and Methods

### 2.1. Binders

Dangote 3× Portland-limestone cement (CEM II B-L, 42.5 N) conforming to NIS 444–1 [20] was used as the main binder. An industrially made pozzolan (MCC) from Pozzolana Cement Plant of the Nigeria Building and Road Research Institute (NBRRI), Ota, Ogun State, Nigeria, served as the focus SCM. RHA, obtained from rice husk calcined to ash at 700 °C for 1 h in an electric furnace and pulverised using a grinding mill at Laboratory of Nigeria Cereals Research Institute, Baddegi near Bida, Niger State, Nigeria, served as the benchmark SCM. The two SCMs were incorporated in powdered form for the different HPC blends vital by the mix design.

### 2.2. Fine Aggregate

River sand used was in air-dry condition with all the grains lesser than 300 µm eliminated employing the sieving method in conformity with the fine aggregate condition for HPC making [21].

### 2.3. Superplasticiser

Masterglenium Sky 504—a polycarboxylic ether (PCE) polymer-based superplasticiser provided by BASF Limited (West Africa)—was utilised as the superplasticiser and dispensed within the producer’s optimum specification of ≤2% by weight of binder (b_wob_).

### 2.4. SAP

SAP marked FLOSET 27CC ≥ 300 µm as defined in a recent publication by Olawuyi et al. [22] at a fixed content of 0.3% b_wob_ was applied for the research.

### 2.5. Water

Potable water conforming to BS EN 1008 [23] obtainable in the concrete laboratory of the Department of Building Technology, Covenant University, Ota, was used for mixing.

### 2.6. The Design Strength of HPC

HPC has characteristic cube strength of 67 MPa following the margin for mix design. The equation can be written as Equation (3):

*f_m_* = *f_c_* + *ks* (3)

where *f_m_* _=_ target mean strength_;_ *f_c_* = the specified characteristic strength; *ks* = the margin, which is a product of *s* = the standard deviation; and *k* = a constant. Table 1 shows the mix compositions of the seven HPC mixtures following the British method of HPC design from where the mortar was extracted using a 4.75 mm standard sieve.

### 2.7. Compressive Strength

The investigation of compressive strength of HPC mortar with MCC or RHA is based on seven levels of SCMs contents (i.e., 0%, 5%, 10%, 15%, 20%, 25%, and 30%), one level of W/B (i.e., 0.3), one level of SAP content (i.e., 0.3% b_wob_), and three levels of curing age (i.e., 2, 3, and 7 days). The test setup followed BS EN 12390–3 [24] and RILEM Technical Recommendation TC14-CPC 4 [25]. A total of 126 40 mm mortar cubes were investigated using the YES-2000 Model digitised Materials Testing Machine by Eccles Technical Engineering Ltd., England with a 2000 kN highest crushing capacity. The compressive strength (*f_c_*) was calculated using Equation (4):

(4) $$f_{c}=\frac{F}{Ac}$$

where *f*_c_ is the compressive strength of HPC mixture (MPa); *F* is the maximum load at failure, kN; *A_c_* is the specimen area (mm).

### 2.8. Degree of Hydration

The degree of hydration of the binary MCC/RHA-based HPC mortars was calculated using the respective SCMs loss on ignition (LOI), and their proportion was made to adjust for their non-evaporable water (𝑤_𝑛_%) as appropriate.

The degree of hydration (α) is then:

(5) $$\alpha =\frac{Wn}{0.23}\times 100$$

where

(6) $$w_{n,}\%=\frac{\mathrm{dried}\;\mathrm{weight}\;\mathrm{of}\;\mathrm{paste}\;-\mathrm{ignited}\;\mathrm{weight}\;\mathrm{of}\;\mathrm{paste}}{\mathrm{ignited}\;\mathrm{weight}\;\mathrm{of}\;\mathrm{paste}\;-\mathrm{loss}\;\mathrm{of}\;\mathrm{ignition}\;\mathrm{of}\;\mathrm{cement}}\times 100$$

(7) $$\mathrm{LOI}\;(\%)=\frac{\mathrm{as}\;\mathrm{recived}\;\mathrm{weight}\;-\mathrm{ignited}\;\mathrm{weight}\;}{\mathrm{as}\;\mathrm{received}\;\mathrm{weight}}\times 100$$

𝑤_𝑛_ (i.e., non-evaporable water) content of the hydrated mortar pastes was established to estimate the degree of hydration, as stipulated in the literature [21]. This result is the variance in the mass quantity of the crushed paste at 950 °C and 105 °C to estimate the degree of hydration (𝛼) because 1 g of anhydrous cement produces 0.23 g of *w*_𝑛_.

### 2.9. Computation of the Gel/Space Ratio of the PC

Firstly, the gel/space ratio of the PC sample was analysed from the degree of hydration and W/B stipulated by [22].

(8) $$Xpc=\frac{2.06vc\propto c\;}{vc\propto c+\frac{w}{B}}$$

*X_pc_* is the gel/space ratio of PC paste, vc is the specific volume of unreacted cement, ∝c is the degree of hydration of cement, and $\frac{w}{B}$ is the original water to binder ratio. In this paper, the PC had the specific gravity of 3.12, corresponding to a specific volume of $(vc)\;is\;\frac{1}{3.12}$ = 0.312. Due to the uncertainty in the stoichiometry for the pozzolanic reaction of fly ash, Lam et al. [15] adopted the reaction between silica fume and calcium hydroxide (Ca (OH)_2_) in hydrated PC. They also agreed that silica fume could simulate the interaction of fly ash or other pozzolans with tricalcium silicate (C_3_S) in PC.

#### 2.9.1. Integrating MCC and RHA SCMs into Powers’ Model as in Equation (7)

Incorporating the MCC and RHA SCMs into Equation (7) is modified to Equations (9) and (10), respectively, according to Lam et al. [15].

(9) $$\mathrm{Xmcc}=\frac{2.06vc\propto c+2.5vmcc\propto mcc\mathrm{MCC}}{vc\propto c+vmcc\propto mccMCC+w}$$

(10) $$\mathrm{Xrha}=\frac{2.06vc\propto c+2.5vrha\propto rha\mathrm{RHA}\;}{vc\propto c+vrha\propto rhaR\mathrm{HA}+w}$$

*X_mcc_* and *X_rha_* are the gel/space ratios of MCC and RHA mortars; vc,vmcc, and vrha are the specific volumes of PC, MCC and RHA, respectively; *∝c,* *∝mcc*, and *∝rha* are the degrees of reaction of PC, MCC, or RHA, respectively; MCC or RHA are the original content of MCC and RHA in the MCC- or RHA-based HPC mortar. W is the weight of water mixed per kg/m^3^ of the MCC- or RHA-blended mix. In this paper, the MCC and RHA had specific gravities of 2.81 and 2.15, respectively, corresponding to vmcc and vrha = $\frac{1}{2.81}$ = 0.356 and $\frac{1}{2.15}$ = 0.465, respectively. In addition, PC paste’s gel/space ratio was analysed based on degrees of hydration taken after *w_n_* content. Therefore, the gel/space ratios of MCC- and RHA-blended HPCs were computed based on the degree of cement hydration that the proposed model analysed.

#### 2.9.2. Air Content Estimation Following Bolomey’s Equation

The air contents in the absence of SAP voids employing a straight weight proportion of demoulded concrete differ from 0.0 to 5.25% as affected by the different HPC mortar blend contents. Therefore, the calculated early-age compressive strength values were adjusted appropriately for the air content to report the impact of MCC/RHA using Equation (11).

(11) $$f_{c}=K_{\mathrm{Bolomey}}\;((1/\frac{w}{c})-0.5)\;\times \;(1-B\;\times \;(a-a_{o}))$$

K_Bolomey_ constant has a uniform value for all blends (i.e., all the HPC mortars are achieved from the same aggregate), the definite air content (% relative to the volume of concrete), and a_0_, a reference air content. Therefore, the value of constant B of Equation (10), corresponding to Hosholt et al. [16] as a rule of thumb, equals 0.04, then paste phase dominates 25% of the concrete volume. On the other hand, the control HPC mortar has paste contents (volume of binder + water) of 28.23%. Therefore, the constant B for the compressive strength relating to Equation (11) was established as 0.035.

#### 2.9.3. Curve Fitting of Compressive Strength and Gel/Space Ratio

The compressive strength of the various early strength mortar cubes was charted against the calculated gel/space ratio for the respective mixtures (control, MCC-, or RHA-based sieved mortar HPCs).

## 3. Results and Discussion

### 3.1. Degree of Hydration and Early Strength of MCC- or RHA-Based HPC Mortar

Table 2 shows the early degree of hydration of MCC- or RHA-based HPC mortar determined using the conventional method reported in the literature [26,27] compared to control in the first 7 days. As shown in Table 2, the degree of hydration increased with hydration time for all MCC-/RHA-blended HPC mortar mixes. At the age of 2 days, the hydration rate remained within the hydration rate of HPC in all mixtures. This behaviour can be linked to the deceleration phase of the cement reaction caused by small particles’ consumption, leaving only large particles to react, lack of space, or lack of water [28]. However, the degree of hydration at the age of 3 days increases slightly for the control but gradually increases with increasing MCC content. However, the degree of hydration for 7 days remains highest in all the MCC-modified mixtures, with MCCC-5 having the highest value. There was also a noticeable gradual decline in the degree of hydration values for MCCC-25 and MCCC-30 mixtures, respectively. These results are largely ascribed to the increasing MCC content in the mixtures. The relative rates of hydration denoted RH_7_ factor (based on the 7th-day degree of hydration for the control mixture) were highest for MCCC-5, as presented in Table 2. Mohsen et al. [29] reported a higher degree of hydration in the hardened cement control sample than the blended cement with SCMs. Moreover, the degree of hydration decreased with increasing the content of SCMs. The result presented here aligns with the literature’s inferences [29]

Table 2 also unveils the degree of hydration for all RHA-blended HPC mortar mixtures with ages. After two days of curing, RHAC-5 to RHAC-30 mixtures obtained the degree of hydration 21.24, 21.90, 21.15, 15.60, 15.21, and 15.33%, respectively. The degree of hydration at 3 days varied between 25.27% to 32.10% for all the mixes. The degree of hydration at 7 days remained highest at 45.47% for the RHAC-10 mixture in this figure. In all cases, RHAC-10 and RHA-15 showed the highest degree of hydration for 2, 3, and 7 curing days. Comparing various HPCs shows that the control mortar produced the highest hydration of 51.30%. The findings reveal that the blend of RHA cement retarded plain cement’s hydration at an early age and confirmed the assertion of Mosaberpanah and Umar [2] on the early resistance to the formation of calcium hydroxide after the initial hydration process after 3 h. The authors view that the increase in the quantity of RHA in cement substitution negatively altered the rate of hydration and the development of the microstructure in the cementitious matrix. The relative rates of hydration denoted RH_7_ factor (based on the 7th-day degree of hydration for control mixture) were highest for RHAC-10, as presented in Table 2.

### 3.2. Early Age Strength Development of HPC Mortar

**Figure 1:**
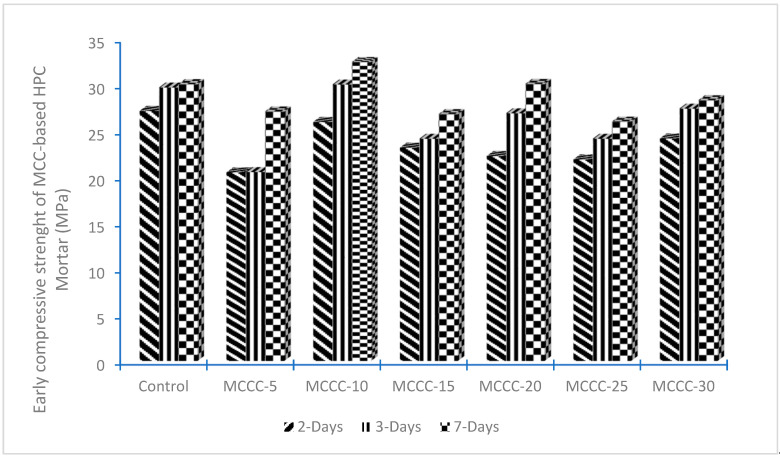
Early compressive strength of MCC-based HPC at different curing days.

The early compressive strength of HPC mortar made with seven different percentage substitutions (0%, 5%, 10%, 15%, 20%, 25%, and 30%) of CEM II with MCC was conducted at 2, 3, and 7 days. The results are given in Figure 1. At up to 30% replacement of CEM II with MCC, only MCCC-10 and MCCC-20 mortars had improved higher strength than the control mortar at all ages. Furthermore, although the compressive strength of the MCCC-5, MCCC-15, MCCC-25, and MCCC-30 mortars with MCC content was lower than CEM II percentages, the differences remain marginal. Therefore, all mixtures with MCC addition had a compressive strength comparative to the control 7 days. For example, the average compressive strengths of HPC mortars containing 0%, 5%, 10%, 15%, 20%, 25%, and 30% MCC measured at 7 days were 30.9, 27.12, 32.50, 26.83, 30.12, 26.02, and 28.35 MPa, respectively. Accordingly, the strength values at 2 days were 27.18, 20.49, 25.98, 23.19, 22.18, 21.91, and 24.19 MPa. It was found that the compressive strength of only MCCC-10 and MCCC-20 mortars increased up to 6.15% and 1.12%, respectively, for 7 days of curing age. Marchetti et al. [4] reported a reduction in the compressive strength of the natural illitic shale of Olavarria, Argentina, calcined at 950 °C blended in concrete at early ages of 2 and 7 compared to the control mixture. Therefore, the authors maintained that higher MCC content in concrete mixtures decreases the material’s mechanical properties. This phenomenon may be attributed to the dilution effect of MCC on the HPC mixture.

**Figure 2:**
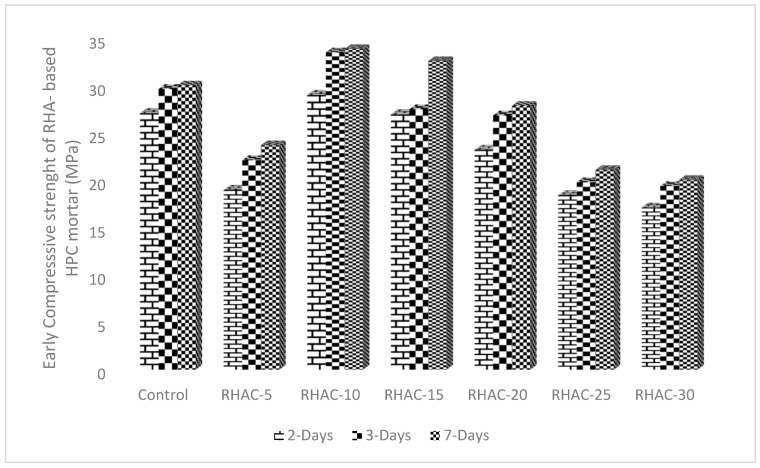
Early compressive strength of RHA-based HPC at different curing days.

The early compressive strength of HPC mortar made with seven different percentage substitutions (0%, 5%, 10%, 15%, 20%, 25%, and 30%) of CEM II with RHA was conducted at 2, 3, and 7 days. The results are given in Figure 2. At up to 30% replacement of PC, with RHA, only RHAC-10 and RHAC-15 mortars had improved higher strength than the control mortar at all ages. Furthermore, although the compressive strength of the RHAC-5, RHAC- 20, RHAC-25, and RHAC-30, mortars with RHA content was lower than PC percentages, the differences remained substantial with RHAC-5, RHAC-25, and RHAC-30. The RHAC-20 HPC mixture had a comparative strength value with the control. For instance, the average compressive strengths of HPC mortars containing 0%, 5%, 10%, 15%, 20%, 25%, and 30% RHA measured at 7 days were 30.9, 23.76, 33.95, 32.67, 27.9, 21.14, and 20.08 MPa, respectively. Accordingly, the strength values at 2 days were 27.18, 19.04, 29.11, 27.05, 23.29, 18.47, and 17.18 MPa. It was found that the compressive strength of only RHAC-10 and RHAC-15 mortars increased up to 11.37% and 7.90%, respectively, for 7 days of curing age. There is usually a delayed compressive strength of RHA-blended cement at an early age under various W/B ratios [30]. This behaviour is occasioned by lowering the cement content that influences the decrease of the hydration products and the lack of calcium hydroxide in the mixture.

### 3.3. Curve-Fitting and Modelling of the Compressive Strength Behaviours

**Figure 3:**
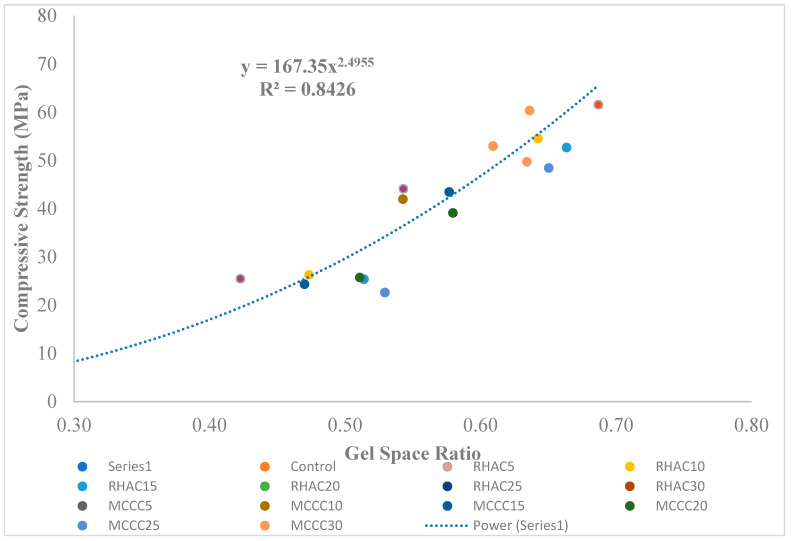
Compressive strength against the gel/space ratio for HPC mortar cubes.

The plot in Figure 3 revealed the association between compressive strength and gel/space ratio and the fitting curve of MCC/RHA mortar. The plot revealed that the compressive strength of mortar cubes (plus or minus MCC or RHA supplement) versus the gel/space ratio inclines to that submitted by the Powers’ model. The correlation inferred from Figure 3 can be stated as shown in Equation (12).

*f_c_* = 167 X^2.50^ (12)

Furthermore, in MCC- and RHA-blended mixtures, the plot showed that the gel/space ratio fits positively with the compressive strength through the Powers exponent equation. However, a reduction in the Powers value could be noticed from the 2.5 value compared with Powers [14] 3 value. The trendline power value of 2.50 is close to 3 as proposed in the Powers’ model, while the constant A is 167. However, it is noted that the R^2^ is 0.84 because of the many factors (binder types, W/B, curing age, and SAP content) combined in the plots. Hasholt et al. [16] reported a related result of 2.88 Powers value and 299 as constant at an R^2^ of 0.97. The factors contributing to the reduction are porosity and the influence of cement-produced gel and SCM-produced gel for a given sample [11]. Wu et al. [11] infer that at early hydration ages of cementitious material when gel/space ratio is minimal, there appears to be relationship inconsistency influenced by porosity. Moreover, cement- and SCM-formed gels for a given sample affect the correlation between gel/space ratio and compressive strength.

## 4. Conclusions

This study analysed the nexus amid the gel/space ratio and early age compressive strength of MCC-/RHA-based HPC mortar and their fitting curves. Fresh mortar extracted from HPC through a 4.75 mm standard sieve was cast in 40 mm^3^ moulds and tested for early compressive strength after curing for 2, 3, and 7 days. Fragments from the compressive strength test of various PC replacements was subjected to a degree of hydration test using the non-evaporable water method. Based on the results, the ensuing deductions can be made.

- The degree of hydration enhances with hydration age for entirely MCC-blended HPC mortar mixes.
- There is a noticeable gradual decline in the degree of hydration values for higher MCC replacements.
- The blend of RHA cement retards plain cement’s hydration at an early age for all HPC mixes.
- There is a comparative early compressive strength of MCC-based HPC mortar with control measured up to 7 days.
- The compressive strength of only RHAC-10 and RHAC-15 mortars improved over the control for 7 days curing age.
- The MCC or RHA addition led to about 17% reduction in the compressive strength of the low W/B HPC.
- The combined use of Bolomey’s void volume correction and Powers gel/space equations are suitable models for predicting the effect of MCC and RHA on the compressive strength of the low W/B HPCs.

## Figures and Tables

**Table 1:** Mix constituents of HPC with MCC.

Constituents	Mix Blends (kg/m^3^)						
	Control	MCCC-5/RHAC-5	MCCC-10/RHAC-10	MCCC-15/RHAC-15	MCCC-20/RHAC-20	MCCC-25/RHAC-25	MCCC-30/RHAC-30
Water	156	156	156	156	156	156	156
Cement (CEM II)	540	513	486	459	432	405	378
MCC	0	27	54	81	108	135	162
Coarse aggregate	1050	1050	1050	1050	1050	1050	1050
Sand (≥300 um)	700	700	700	700	700	700	700
SAP (0.3% b_wob)_	1.62	1.62	1.62	1.62	1.62	1.62	1.62
Superplasticiser (1.5% b_wob_)	8.10	8.10	8.10	8.10	8.10	8.10	8.10
Water/binder (W/B)	0.3	0.3	0.3	0.3	0.3	0.3	0.3
Additional water	20.30	20.30	20.30	20.30	20.30	20.30	20.30

**Table 2:** Influence of MCC or RHA binder types on the degree of hydration of HPC mortar.

	Degree of Hydration (%)			RH_7_ Factor		
MIX ID	2 Days	3 Days	7 Days	2 Days	3 Days	7 Days
Control	30.79	32.67	51.30	0.60	0.64	1.00
MCCC-5	28.99	38.40	50.29	0.57	0.75	0.98
MCCC-10	20.87	34.98	44.15	0.41	0.68	0.86
MCCC-15	20.30	33.46	36.22	0.40	0.65	0.71
MCCC-20	20.45	32.61	41.47	0.40	0.64	0.81
MCCC-25	18.85	30.26	40.62	0.37	0.59	0.79
MCCC-30	18.79	31.58	33.42	0.37	0.62	0.65
RHAC-5	21.24	29.24	36.77	0.41	0.57	0.72
RHAC-10	21.90	31.86	45.47	0.43	0.62	0.89
RHAC-15	21.15	32.10	43.89	0.41	0.63	0.86
RHAC-20	15.60	25.27	33.84	0.30	0.49	0.66
RHAC-25	15.21	23.37	33.07	0.30	0.46	0.64
RHAC-30	15.33	21.60	32.21	0.30	0.42	0.63

## Data Availability

Not applicable.
